# Identification and Characterization of Five BAHD Acyltransferases Involved in Hydroxycinnamoyl Ester Metabolism in Chicory

**DOI:** 10.3389/fpls.2016.00741

**Published:** 2016-06-06

**Authors:** Guillaume Legrand, Marianne Delporte, Chahinez Khelifi, Adeline Harant, Christophe Vuylsteker, Monika Mörchen, Philippe Hance, Jean-Louis Hilbert, David Gagneul

**Affiliations:** Agro-Food and Biotechnology Research Institute, EA 7394, Charles Viollette Research Institute, Université de Lille 1Villeneuve d’Ascq, France

**Keywords:** caffeic acid esters, BAHD family, acyltransferases, chlorogenic acid, chicory (*Cichorium intybus*), functionally redundant genes

## Abstract

Chicory (*Cichorium intybus*) accumulates caffeic acid esters with important significance for human health. In this study, we aim at a better understanding of the biochemical pathway of these bioactive compounds. Detailed metabolic analysis reveals that *C. intybus* predominantly accumulates caftaric and chicoric acids in leaves, whereas isochlorogenic acid (3,5-diCQA) was almost exclusively accumulated in roots. Chlorogenic acid (3-CQA) was equally distributed in all organs. Interestingly, distribution of the four compounds was related to leaf age. Induction with methyljasmonate (MeJA) of root cell suspension cultures results in an increase of 3-CQA and 3,5-diCQA contents. Expressed sequence tag libraries were screened using members of the BAHD family identified in *Arabidopsis* and tobacco as baits. The full-length cDNAs of five genes were isolated. Predicted amino acid sequence analyses revealed typical features of BAHD family members. Biochemical characterization of the recombinant proteins expressed in *Escherichia coli* showed that two genes encode HCTs (hydroxycinnamoyl-CoA:shikimate/quinate hydroxycinnamoyltransferases, HCT1 and HCT2) whereas, three genes encode HQTs (hydroxycinnamoyl-CoA:quinate hydroxycinnamoyltransferases, HQT1, HQT2, and HQT3). These results totally agreed with the phylogenetic analysis done with the predicted amino acid sequences. Quantitative real-time polymerase chain reaction analysis of gene expression indicated that HQT3, HCT1, and HCT2 might be more directly associated with CQA accumulation in cell culture in response to MeJA elicitation. Transient expression of HCT1 and HQT1 in tobacco resulted in a higher production of 3-CQA. All together these data confirm the involvement of functionally redundant genes in 3-CQA and related compound synthesis in the *Asteraceae* family.

## Introduction

Plants accumulate a wide range of specialized metabolites with a large diversity of chemical types. Phenolic compounds are recognized for their health benefit effects and are the most important dietary antioxidants. They have drawn increasing attention due to their marked effect in the prevention of various oxidative stress associated diseases. Among them, hydroxycinnamic acid derivatives, particularly hydroxycinnamoyl esters, are widely distributed in the plant kingdom (Petersen et al., 2009). For example, a caffeoyl moiety can be combined to quinic acid to form either monocaffeoylquinic acid (chlorogenic acid, CQA) as found in tobacco (*Nicotiana tabacum* L.*)* or in tomato (*Solanum lycopersicum* L.; Niggeweg et al., 2004) or dicaffeoylquinic acid (isochlorogenic acid, diCQA) as in coffee (*Coffea* spp.), tomato or sweet potato (*Ipomea batatas* L.; Kojima and Kondo, 1985; Lallemand et al., 2012; Moglia et al., 2014). In lettuce (*Lactuca sativa* L.) or red clover (*Trifolium pratense* L.), the caffeoyl group is attached to malic acid to form phaselic acid (Sullivan, 2009; Mai and Glomb, 2013). Esters of tartaric acid such as monocaffeoyltartaric acid [caftaric acid (CTA)] are also found in grape vine (*Vitis vinifera* L.), perennial peanut (*Arachis glabrata* L.) and several members of the *Asteraceae* like purple coneflower (*Echinacea purpurea* L.) which also contains dicaffeoyltartaric acid (chicoric acid, diCTA; Singleton et al., 1986; Perry et al., 2001; Sullivan and Foster, 2013). Chicory (*Cichorium intybus* L.) is a member of the *Asteraceae* family used for a long time in traditional medicine. This is notably due to the accumulation of high-value health promoting compounds such as CQA, diCQA, CTA, and diCTA (Kandeler and Ullrich, 2009; Bahri et al., 2012). Indeed, many health benefit effects are attributed to these molecules when isolated (Tousch et al., 2008; Koriem and Soliman, 2014; Yasir et al., 2016). In addition to their notable dietary role, these molecules are important compounds with multiple roles in plants. They are involved in plant protection against abiotic (UV, oxidative stress) and biotic (insects, pathogens) stresses. Indeed, in *V. vinifera*, CTA accumulation was shown to be related to the presence of UV radiation supporting a putative role of this molecule in UV protection (Del-Castillo-Alonso et al., 2014). In globe artichoke (*Cynara cardunculus* L.), it was also hypothesized that diCQA could play a role in UV protection (Moglia et al., 2008). Increased CQA accumulation in *S. lycopersicum* was shown to improve antioxidant activity and resistance to infection by *Pseudomonas syringae* (Niggeweg et al., 2004). Furthermore, CQA as well as feruloylquinate were also identified as protective agents against thrips in chrysanthemum (*Dendranthema grandiflora*; Leiss et al., 2009).

**FIGURE 1:**
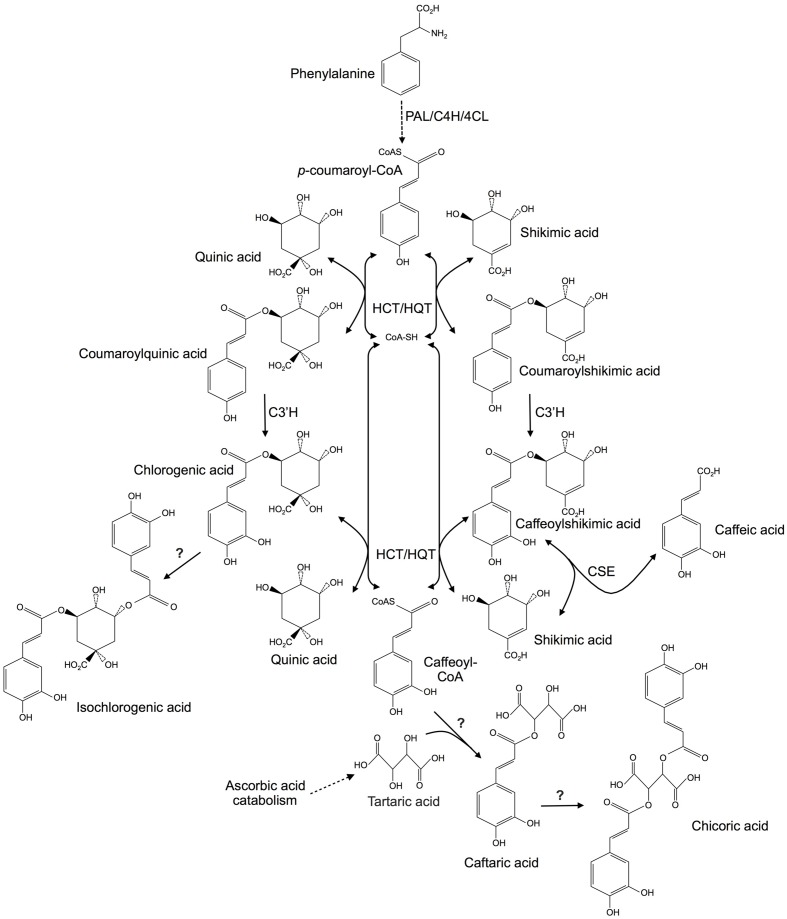
**Putative metabolic pathways involved in hydroxycinnamic acid biosyntheses in plants**. Question marks indicate enzymes that have been characterized biochemically but which are not supported by molecular data. PAL, phenylalanine ammonia lyase; C4H, cinnamate 4-hydroxylase; 4CL, 4-coumaroyl CoA ligase; HCT, hydroxycinnamoyl-CoA:shikimate/quinate hydroxycinnamoyltransferase; HQT, hydroxycinnamoyl-CoA:quinate hydroxycinnamoyltransferase; C3′H, *p*-coumaroyl ester 3′-hydroxylase; CSE, caffeoyl shikimate esterase.

A better understanding of CQA, diCQA, CTA, and diCTA metabolic pathways is of paramount importance to develop agronomic, genetic, or biotechnological tools for higher production of theses high-value bioactive compounds. In this context, *C. intybus* could be a model species considering that the metabolism of these molecules is highly interconnected (**Figure 1**). The aromatic parts of the esters are synthesized *via* the phenylpropanoid pathway. Most enzymes involved in the first part of this pathway are known for years (Vanholme et al., 2010). The entry point is the aromatic amino acid phenylalanine (Phe) arising from the shikimate pathway. Deamination of Phe by Phe ammonia lyase (PAL) leads to cinnamic acid. Cinnamate-4-hydroxylase and 4-coumarate coenzyme A (CoA) ligase (4CL) generate *p*-coumaroyl-CoA from cinnamic acid. Thereafter, hydroxycinnamoyltransferases (HCTs) convert the CoA-thioester to coumaroylquinate or coumaroylshikimate which is subsequently hydroxylated by *p*-coumarate-3′-hydroxylase (C3′H) to form the caffeoyl derivatives. Two types of transferases have been identified. Hydroxycinnamoyl-CoA: shikimate/quinate HCTs use shikimate as a preferred acyl acceptor whereas hydroxycinnamoyl-CoA: quinate hydroxycinnamoyltransferases (HQTs) exhibit preference for quinate (Lallemand et al., 2012). Experimental evidence has shown that HCTs may be involved in the synthesis of precursors for lignin synthesis whereas HQTs may be directly involved in CQA synthesis toward its accumulation (Hoffmann et al., 2003; Niggeweg et al., 2004). HQT and HCT activities are fully reversible: quinate or shikimate derivatives can be converted in presence of CoA to the free acid plus *p*-coumaroyl-CoA or caffeoyl-CoA. Recently, a caffeoylshikimate esterase (CSE) that releases caffeic acid and shikimate has been identified in *Arabidopsis* (**Figure 1**; Vanholme et al., 2013). HCTs and HQTs belong to the BAHD superfamily of plant-specific acyl-CoA dependent acyltransferases (St-Pierre and De Luca, 2000; D’Auria, 2006; Yu et al., 2009; Tuominen et al., 2011). In *I. batatas*, diCQA arises from the transfer of a caffeoyl group from a CQA to a second CQA (Villegas et al., 1987). An enzyme called chlorogenic acid: chlorogenate caffeoyl transferase catalyzes this reaction. The protein was partially characterized but the corresponding gene has not been identified (Villegas et al., 1987). Recent study identified a HCT from *Coffea canephora* able to convert CQA to diCQA (Lallemand et al., 2012). A *S. lycopersicum* HQT was also shown to be involved in the formation of diCQA from CQA (Moglia et al., 2014). In *A. glabrata* and *Equisetum arvense*, it was shown that CTA is synthesized through condensation of a caffeoyl-CoA with tartaric acid which is a typical mechanism of the BAHD family members (Hohlfeld et al., 1996; Sullivan, 2014). Furthermore chicoric acid was demonstrated *in vitro* to be synthesized by transfer of a caffeoyl moiety of caffeoyl-CoA to CTA in *E. arvense* (Hohlfeld et al., 1996). Pathways involved in CTA and diCTA are only supported by biochemical experiments and to date, no molecular data are available. To summarize, all these molecules could arise through the action of acyltransferases of the BAHD family.

In chicory, knowledge on the genetic control of these metabolic pathways is still limited. We report in this paper the isolation, the cloning, and the biochemical and functional characterization of five full-length cDNA sequences encoding HCTs or HQTs in *C. intybus*. The properties of these enzymes are consistent with a role in CQA production and detailed analysis confirms that both types of enzymes are subsequently needed to ensure CQA synthesis and accumulation.

## Materials and Methods

### Plant Material and Growth Conditions

Seeds of chicory, *C. intybus* L. var Orchies, were provided by Florimond-Desprez (Cappelle en Pévèle, France). They were germinated for 7 days in Petri dishes on paper humidified with half strength MS medium at pH 6.0 in a growth chamber (Photoperiod, 16 h: 8 h, light: dark; thermoperiod, 24°C: 18°C, day: night; Murashige and Skoog, 1962). The 7 days-old seedlings were then transferred hydroponically to culture pots containing half strength MS solution. The volume of the solution was daily adjusted with fresh medium and the medium completely renewed weekly. For analysis of HCT and HQT mRNA levels, for hydroxycinnamoyl ester contents and for full length cDNA cloning, either 6 or 4 weeks-old seedlings were individually harvested. Three biological replicates were sampled. Roots and leaves were separately collected and for the 4 weeks-old seedlings, leaves were divided in three samples: old leaves, medium age leaves (intermediate leaves) and newly expanding leaves (young leaves). Samples were immediately frozen in liquid nitrogen and stored at -80°C until needed. Chicory cell cultures were prepared as described in Delporte et al. (2015).

### Isolation of Full-Length cDNA Sequences

Putative BAHD members of clade Vb were identified by searching publicly available databases with AtHCT and NtHQT. Full-length cDNAs of CiHCT1, CiHCT2, CiHQT1, CiHQT2, and CiHQT3 were first subcloned into a TA cloning vector (pGEM-T easy, Promega). DNA sequences were amplified by PCR using proofreading polymerase (PrimeSTAR HS DNA polymerase, Takara). The PCR primers designed for these amplifications are listed in Supplementary Table S1. The sequences of independent clones were determined in their entirety on both strands and the consensus sequences established. One clone per gene was kept and was used for following cloning (pGEMT-easy-HCT1, pGEMT-easy-HCT2, pGEMT-easy-HQT1, pGEMT-easy-HQT2, and pGEMT-easy-HQT3).

### Phylogenetic Tree of Acyltransferases and Multiple Sequence Alignment

Hydroxycinnamoyltransferases cDNAs from chicory were translated to the corresponding amino acid sequences. Phylogenetic tree and multiple alignments were generated by CLC Sequence Viewer 7 software.

### Expression, Purification, and *In Vitro* Assays of Recombinant Proteins

Full-length cDNAs of the five genes were amplified using a proofreading polymerase (PrimeSTAR HS DNA polymerase, Takara) using the previously mentioned sequenced clones (pGEMT-easy-HCT1, 2 and pGEMT-easy-HQT1, 2, or 3) as the templates and the primers listed in Supplementary Table S1. The entry clones (pDONR221-HCT1 or 2 and pDONR221-HQT1, 2, or 3) were obtained through recombination of the PCR products with pDONR221 (Invitrogen). For further expression analysis, the open reading frames were introduced into pDEST17 expression vector (Invitrogen) by LR recombination to produce PDEST17-HCT1 or 2 and pDEST17-HQT1, 2, or 3. Recombinant proteins with an N-terminal 6xHis-tag were expressed in *Escherichia coli* BL21-CodonPlus-(DE3)-RIL cells following induction with 1 mM isopropyl β-D-1-thiogalactopyranoside. After induction, cells were harvested by centrifugation and pellets stored at -80°C. Cell pellets were resuspended in the extraction buffer (50 mM NaH_2_PO_4_, 500 mM NaCl, 20 mM imidazole pH 7.4) added with 1 mg ml^-1^ lysozyme and the suspensions were incubated on ice for 30 min. After sonication on ice, samples were centrifuged (10000 *g*, 4°C, 30 min) and the supernatants were loaded onto 1 ml HisTrap HP columns (GE Healthcare) and processed according to the manufacturer’s procedures. The positive fractions were identified by SDS-PAGE, pooled, desalted with a PD-10 desalting column (Pharmacia), and concentrated using vivaspin sample concentrators with 30 kDa molecular mass cutoff (GE Healthcare). Purified proteins were stored at -20°C until needed.

The standard enzymatic *in vitro* assay was performed in a volume of 50 μL containing acyl donor (hydroxycinnamoyl-CoA) and acyl acceptor (shikimate or quinate) at various concentrations, 0.1–1 μg of purified enzyme and 50 mM phosphate buffer pH 7.1. The reactions were incubated at room temperature and stopped by adding 10 μl formic acid 10%. The reaction mixture were then filtered through a 0.45 μm filter (Pall GHP, VWR) and analyzed by HPLC-UV. K_m_ and V_max_ values were determined in triplicates by fitting Michaelis-Menten curves directly using GraphPad-Prism Software. Saturating concentration of acyl-CoAs was set at 800 μM. For evaluation of the optimal pH, the reaction mixture was incubated at room temperature in 50 mM phosphate buffer at pH ranging from 5.0 to 9.0 or in 50 mM acetate buffer at pH ranging from 4.0 to 5.0. Reactions were run in triplicates.

### Enzymatic Production and Purification of Hydroxycinnamoyl-CoA Compounds

*p*-coumaroyl-CoA, caffeoyl-CoA, feruloyl-CoA, and cinnamoyl-CoA were prepared enzymatically from each acid and purified (Beuerle and Pichersky, 2002; Rautengarten et al., 2010). Purified *Nicotiana benthamiana* 4-coumarate: CoA ligase 1 (Nb4CL1) recombinant protein from *E. coli* was used to synthesized the CoA-thioesters following the procedure described by Rautengarten et al. (2010). The plasmid for protein expression was kindly provided by Pr. Sheller (Emeryville, CA, USA). The CoA-thioesters were then purified using a SPE cartridge (Beuerle and Pichersky, 2002). Yields were 97, 90, 95, and 87% for caffeoyl-CoA, *p*-coumaroyl-CoA, feruloyl-CoA, and cinnamoyl-CoA, respectively.

### gDNA Extraction, Amplification, and Sequencing

gDNA was extracted from fully expanded leaves using the Nucleospin Plant II kit (Macherey-Nagel). DNA sequences were amplified by PCR using proofreading polymerase (PrimeSTAR HS DNA polymerase, Takara). The PCR primers designed for these amplifications are listed in Supplementary Table S1. PCR products were sequenced to determine intron sequences and sizes.

### RNA Extraction and cDNA Synthesis

Total RNA was isolated from the different parts of young seedlings and from culture cells as described in Delporte et al. (2015). The yield and purity of total RNA were determined using the Experion Automated Electrophoresis System (Bio-Rad). One μg of DNAse treated total RNA was used for cDNA synthesis with the Reverse Transcriptase Superscript III RNAse H kit and oligo(dT)_20_ primer (Invitrogen).

### qRT-PCR Analysis

Gene specific primers (listed in Supplementary Table S1) were designed using Primer 3 software using recommended para-meters (Udvardi et al., 2008; melting temperature = 60 ± 1°C; length of 18–25 nucleotides; 40–60% GC; length of amplicon from 60 to 150 bp). qRT-PCR was carried out using iQ SYBR Green Supermix (Bio-Rad). Reactions were set up in a 20 μl total volume containing cDNA equivalent to 100 ng of total RNA and 5 μM of each primer. Signals were normalized using geometric mean of *CLATH* and *SAND* mRNA levels for seedling experiments and of *TIP41* and *PP2AA2* mRNA levels for cell culture experiments as described by Delporte et al. (2015). Initial denaturation was 95°C for 3 min followed by 40 cycles including 95°C for 10 s and 60°C for 30 s. A standard dissociation protocol was run at the end of each run to ensure that each amplicon was a single product. Each reaction was run in duplicates. Control PCRs were run with non retro-transcribed RNA to check for gDNA contamination. Calculation of relative expression was done according to the Pffafl equation (Pfaﬄ, 2001).

### Determination of Protein Concentrations, SDS-PAGE Analysis, and Immunoblot Analysis

Protein concentrations of the extracts were determined using Bio-Rad Protein Assay using bovine serum albumin as a standard. SDS-PAGE and immunoblot analysis was conducted as described elsewhere (Sambrook et al., 1989). The following antibody combination were used for immunodetection: penta-His antibody (Qiagen)/alkaline phosphatase (AP)-conjugate anti-mouse IgG (Promega). To estimate molecular masses, the Precision Plus Protein All blue Standards ladder (Bio-Rad) was used.

### Transient Expression in *N. benthamiana*

Previously obtained clones pGEMT-easy-HCT1 and pGEMT-easy-HQT1 were used to amplify full-length cDNA of *HCT1* and *HQT1* with the primers described in Supplementary Table S1. Each PCR product was introduced into pDONR221 by recombination. Open reading frame were then introduced into the expression vector pB2GW7 by LR recombination (Karimi et al., 2002). Empty pB2GW7 was also generated by first cutting the vector with EcoRV (NEB) to remove the RfA containing the ccdB gene. Then, the empty vector was re-circularized using T4 DNA Ligase (NEB). The resulting vector pB2GW7-HCT1, pB2GW7-HQT1, empty pB2GW7 as well as the pEAQ-HT vector, harboring the gene encoding the silencing inhibitor protein p19 (Sainsbury et al., 2009), were individually introduced into the *Agrobacterium tumefaciens* strain GV2260 by electroporation. These four recombinant strains were grown overnight in YEB medium at 28°C, with shaking at 200 rpm. Thereafter, the cells were pelleted by centrifugation at 5000 *g* for 15 min and resuspended in activation buffer containing 10 mM MgCl_2_ and 150 μg ml^-1^ acetosyringone before incubation at room temperature for 3 h. For agroinfiltration, the strain containing the pEAQ-HT was used alone or combined with strain transformed with either pB2GW7-HQT1 or pB2GW7-HCT1 or empty pB2GW7. Whatever the case, final OD_600_ was 0.8 (0.3 pEAQ-HT + 0.5 of the other construct). Each construct or construct combination was used to infiltrate the abaxial air space of two individual leaves per plant. Four 6 weeks-old plants per construct were used. After 4 days, the infiltrated leaves were collected and immediately frozen in liquid nitrogen before freeze-drying.

### HPLC Analysis of Polyphenols

The lyophilized plant material was powdered and 50 mg were resuspended in 1 ml of a methanol/water/acetic acid mixture (75/23/2, v/v/v). The mixtures were then incubated under agitation for 12 h at 4°C. Homogenates were clarified by centrifugation (14000 *g*, 4°C, 10 min) and 300 μl of supernatant were transferred in a new tube. After addition of 150 μl chloroform and 150 μl water, the suspensions were thoroughly shaken and centrifuged (14000 *g*, 5 min, 20°C). The upper phase was collected and passed through a 0.45 μm filter and 5 μl aliquots were analyzed on a 100 mm × 4.6 mm Kinetex 2.6 μm PFP 100 Å column (Phenomenex). For determination of phenolics contents in chicory and tobacco and for kinetics parameter of the enzymes, the chromatographic separation was performed using water (solvent A) and acetonitrile (solvent B) both acidified with 0.1% ortho-phosphoric acid. The solvents were delivered at a flow rate of 1.1 ml min^-1^ and the oven temperature was set at 45°C with start condition at 10% solvent B. The HPLC conditions were as follow: 7 min gradient to 30% solvent B followed by 3 min gradient to 70% and 1.5 min of isocratic 70% solvent B. Then, 1 min gradient to return to 10% solvent B and 8.5 min of isocratic re-equilibration at 10% solvent B. Phenolics were characterized by cochromatography of pure synthetic compounds and quantified making reference to individual external calibration curves at 320 nm.

### Statistics

Statistical analysis were conducted using R 3.2.2 for Mac and used to determined between extract variation for both chicory and tobacco extract (R Core Team, 2015). In accordance with need, ANOVA or Student’s test were used.

## Results

### Profiling of the Main Caffeic Acid Esters in Chicory Tissues

**FIGURE 2:**
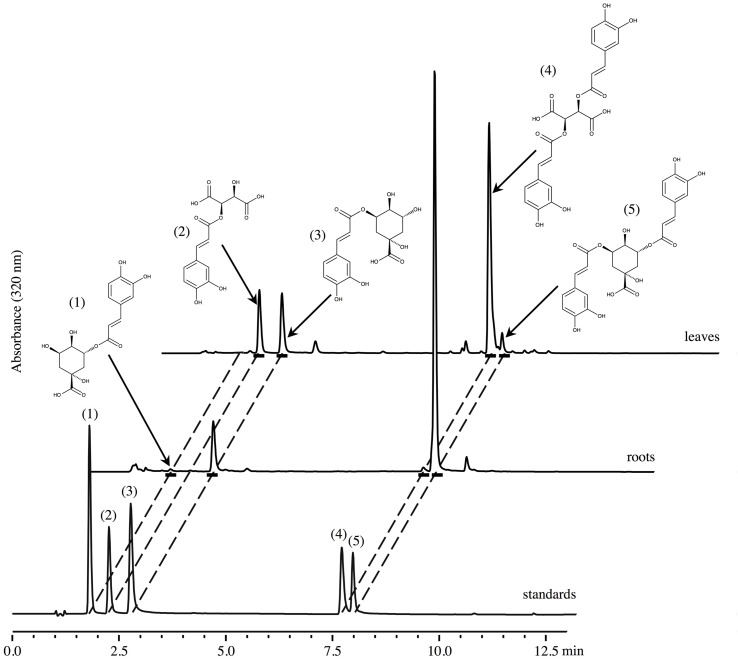
**Chromatograms of crude methanolic extracts from leaves and roots of chicory seedlings and of a standard mixture**. Products were characterized by their retention times and UV absorbance spectra recorded with a photodiode array detector. Standards: (1) neochlorogenic acid, 5-CQA; (2) caftaric acid; (3) chlorogenic acid, 3-CQA; (4) chicoric acid; (5) isochlorogenic acid, 3,5-diCQA.

**FIGURE 3:**
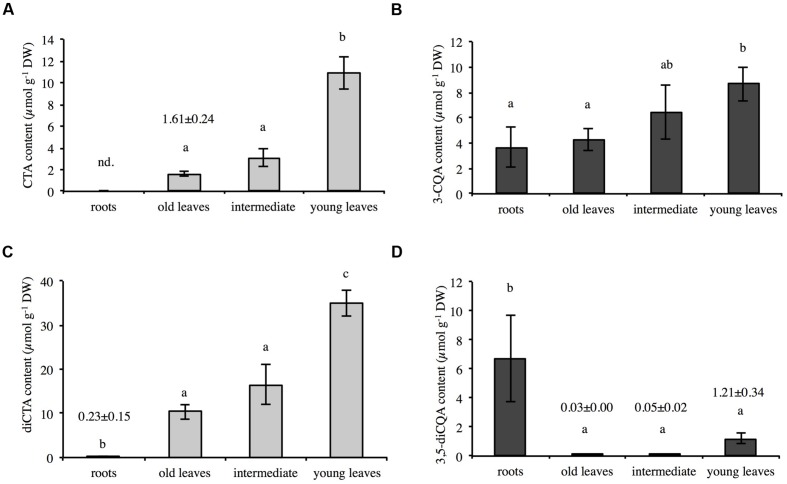
**Contents of CTA (A), 3-CQA (B), diCTA (C), and 3,5-diCQA (D) in roots and leaves (different ages) of chicory seedlings**. Methanolic extracts of roots or leaves of 4-weeks-old seedlings were analyzed by HPLC. The data represent the mean values (±SD) of four biologically independent experiments. Different letters in the same histogram indicate significant differences detected using ANOVA (*P* < 0.05). nd, not detected.

The amounts of CQA, diCQA, CTA, and diCTA were first determined in 6 weeks-old chicory seedlings grown hydroponically in half strength MS medium (Murashige and Skoog medium). Methanolic extracts of roots or leaves were analyzed by HPLC-UV. Typical chromatograms are presented in **Figure 2** and quantifications indicated in Supplementary Table S2. Notably, only trace amounts of diCTA and no CTA could be detected in roots whereas diCQA was poorly abundant in leaves. CQA, a putative precursor of all three other molecules, did not allow to discriminate between organs. In roots, diCQA was the most abundant compound (6.7 ± 3.0 μmol g^-1^ DW, i.e., 62.6% of total caffeate derivatives) while in leaf, diCTA was the most abundant (18.9 ± 2.2 μmol g^-1^ DW, i.e., 63.4% of total caffeate derivatives). Detailed analyses show that chicory predominantly accumulates one of the CQA isomers, 3-CQA (i.e., chlorogenic acid according to the actual CAS nomenclature), and 3,5-diCQA one of the diCQA isomers. However, 5-CQA (i.e., neochlorogenic acid, actual CAS nomenclature) could also be detected in trace amount in chicory roots but not in leaves. Overall leaf phenolic contents were about three times higher than those in roots (29.8 and 10.7 μmol g^-1^ DW, respectively). To extend our analysis, leaves of 4 weeks-old seedlings were separately collected and analyzed. Interestingly, levels of CTA, diCTA, 3-CQA, and 3,5-diCQA were dependent on leaf ages (**Figure 3**). The highest contents were found in the youngest leaves. For instance, CTA content was seven times higher in the youngest leaves than in the older ones (**Figure 3A**).

### Modulation of Caffeic Acid Ester Contents by MeJA Elicitation in Root Culture Cells

**FIGURE 4:**
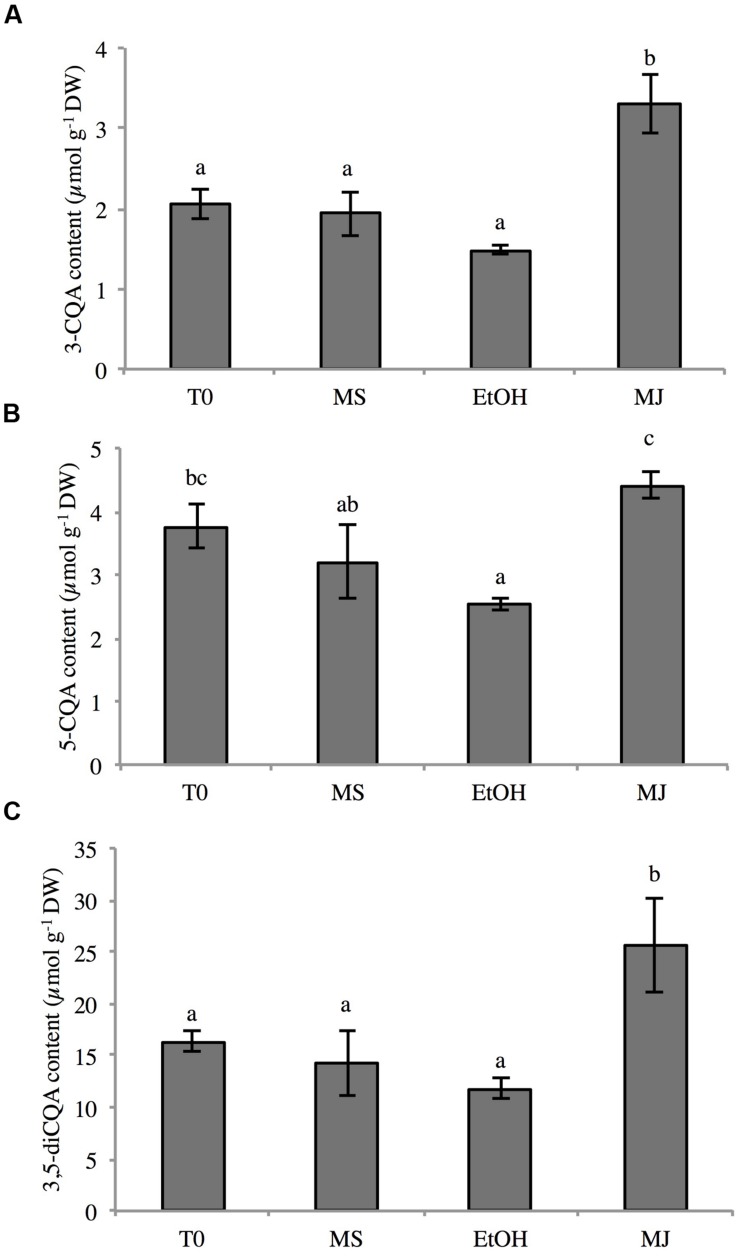
**Contents of 3-CQA (A), 5-CQA (B), and 3,5-diCQA (C) in chicory cell cultures under different conditions**. Methanolic extracts were analyzed by HPLC. T0: cell culture sampled before elicitation, MS: cultures kept on MS medium for 24 h, EtOH: cell cultures treated with ethanol for 24 h, MJ: cell cultures treated with 50 μM MeJA for 24 h. Values are means of three replicates ± SD. Different letters in the same histogram indicate significant differences detected using ANOVA (*P* < 0.05).

As an inducer of jasmonates, methyljasmonate (MeJA) regulates a diverse set of physiological and developmental processes. It has been observed that addition of MeJA can significantly alter the production of specialized metabolites (De Geyter et al., 2012; Wasternack and Hause, 2013). Phenylpropanoid compounds were shown to accumulate in *N. tabacum* cell cultures upon MeJA elicitation (Gális et al., 2006). In our lab, we have shown that elicitation of chicory cell cultures with MeJA readily enhances 3-CQA and 3,5-diCQA production and accumulation (unpublished results). Thus, chicory root cell cultures were treated for 24 h with 50 μM MeJA diluted in ethanol (EtOH) and the contents of 3-CQA, 5-CQA, and 3,5-diCQA were analyzed (**Figure 4**). As expected, upon addition of MeJA, the concentrations of 3-CQA and 3,5-diCQA were 2.2 fold higher than in the control cells grown in the presence of the sole ethanol. The levels of 3-CQA and 3,5-diCQA were 1.5 and 11.8 μmol g^-1^ DW, respectively in the cells treated with EtOH and 3.3 and 25.6 μmol g^-1^ DW in the cells elicited with MeJA. As previously described in chicory roots, 5-CQA was also detected in this material but, surprisingly, in quantity quite similar to that of 3-CQA (**Figure 4**). Despite a variation in 5-CQA quantity less pronounced than these of the other quantified phenolics, significant difference between elicited cells and cells treated with EtOH is notable (4.4 and 2.5 μmol g^-1^ DW respectively). No CTA or diCTA could be detected in this suspension culture. This is in agreement with the seedling data.

### Identification, Cloning, and Sequence Analysis of Five Hydroxycinnamoyl Transferase Genes in Chicory

To identify sequences of putative members of the BAHD family from chicory, the amino acid sequences of HCT from *Arabidopsis thaliana*, i.e., AtHCT (GenBank accession AED95744) and of HQT from *N. tabacum*, i.e., NtHQT (GenBank accession CAE46932) were used as the queries in a tBLASTn search of the EST sequences publicly available. The ESTs showing the highest similarity to either AtHCT or NtHQT were selected for further analysis. This search identified 15 ESTs (GenBank accessions EH696409, FL673648, DT213617, EH686422, EH674585, EH692212, FL682645, EH691266, EH677208, EH692526, EH705216, EH706054, EH682505, EH692394, and EH701989) that were further assembled in four contigs. According to the homology of the predicted aminoacid sequences to either the AtHCT or NtHQT, they were named *HCT1*, *HCT2* (GenBank accession KT222892), *HQT1* (GenBank accession KT222893), and *HQT2* (GenBank accession KT222894). Examination of predicted amino acid sequences showed that *HCT2*, *HQT1*, and *HQT2* were the full-length cDNA. No additional overlapping ESTs were identified to extend the *HCT1* sequence. For this reason, a chicory bacterial artificial chromosome library was screened and one positive clone was identified (Gonthier et al., 2010). The 5′ sequence was extended by sequencing to get the full-length sequence (GenBank accession KT222891). To get more putative sequences, the recently released 454 reference assemblies of *C. intybus* was interrogated and an additional HQT, *HQT3* (comp5746_C0-seq1, GenBank accession KT222895), was identified (Hodgins et al., 2014).

The *HCT1* coding region shares 81% nucleotide sequence identity with that of *HCT2*. *HQT2* coding region shares 74 and 66% nucleotide sequence identity with that of *HQT3* and *HQT1*, respectively. *HQT1* and *HQT3* share 67% nucleotide sequence identity. Comparison of *HCT* coding region with that of *HQT* gave a maximum score of 61%. Gene sizes were 1305, 1293, 1317, 1320, and 1326 bp for *HCT1*, *HCT2*, *HQT1*, *HQT2*, and *HQT3*, respectively. *HCT1* encodes a protein of 434 amino acids with a calculated molecular mass of 48.1 kDa. The peptide product of *HCT2* consists of 430 amino acid residues and has a predicted molecular mass of 47.7 kDa. The 438-amino acid protein encoded by *HQT1* has a predicted molecular mass of 48.6 kDa. *HQT2* and *HQT3* encode proteins of 439 and 441 amino acids, respectively, with calculated molecular masses of 48.6 and 48.8 kDa.

**FIGURE 5:**
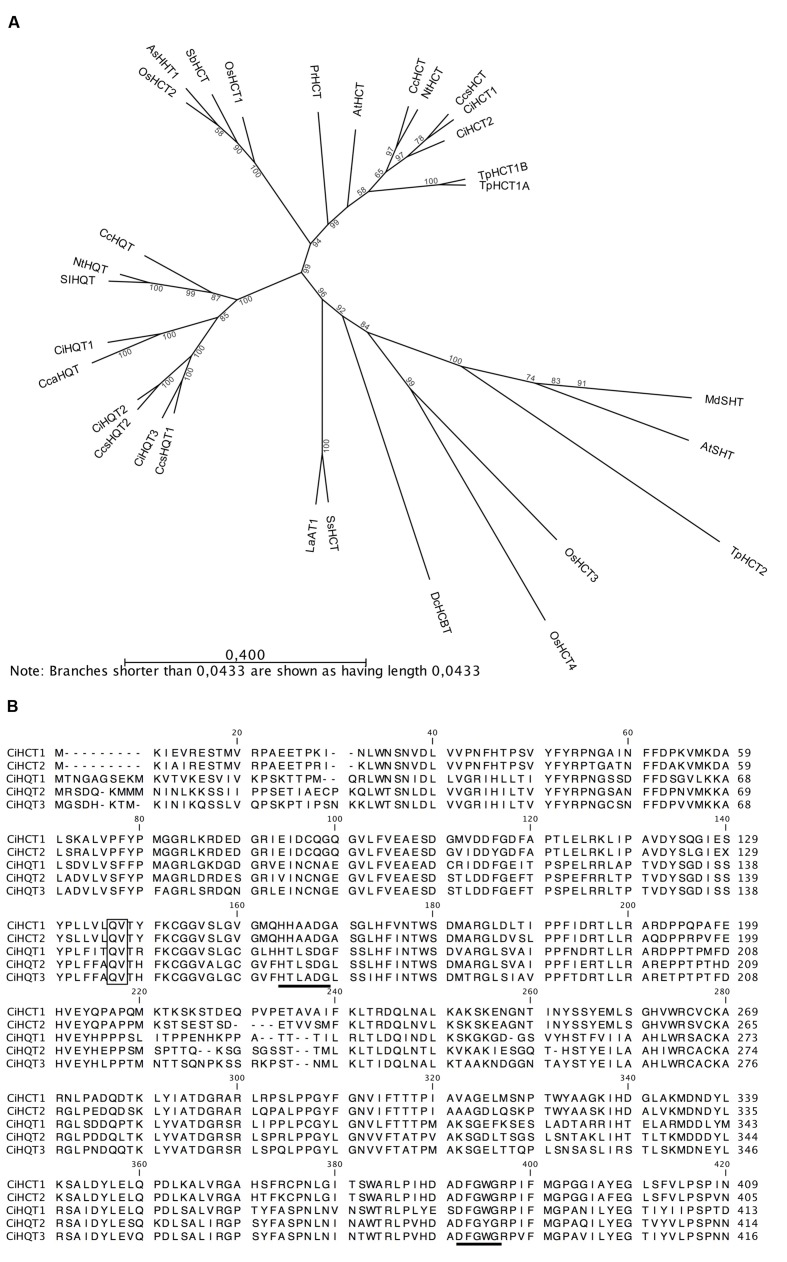
**Phylogenetic analysis and sequence comparison of the five members of the BAHD family identified in this study. (A)** Phylogenetic tree based on the amino acid sequences of HCTs and HQTs from chicory and biochemically characterized members of BAHD family members. Only the clade Vb is represented. The phenogram was generated by the neighbor-joining method following alignment by the MUSCLE algorithm (with default parameters) using CLC Sequence Viewer 7 software (bootstrap method, 500 replications). The length of the line indicates the relative distance between nodes thanks to the scale bar provided. **(B)** Amino acid sequence comparison of chicory HCTs and HQTs. Black bars indicate the conserved BAHD acyltransferase domains (HXXXDG and DFGWG). The intron “Q” when present is located between the Q and V residue of the predicted aminoacid sequence (black frame). CcsHCT: HCT from *Cynara cardunculus* var. *scolymus* (AFL93686), CiHCT1: this work (KT222891), CiHCT2: this work (KT222892), NtHCT: HCT from *Nicotiana tabacum* (CAD47830), CcHCT: HCT from *Coffea canephora* (CAJ40778), TpHCT1A: HCT from *T. pratense* (ACI16630), TpHCT1B: HCT from *T. pratense* (ACI28534), AtHCT: HCT from *Arabidopsis thaliana* (AED95744), PrHCT: HCT from *Pinus radiata* (ABO52899), SbHCT: HCT from *Sorghum bicolor* (4KE4_A), OsHCT1: HCT from *Oryza sativa* (NP_001053225.1), OsHCT2: HCT from *O. sativa* (NP_001047408.1), AsHHT1: hydroxyanthranilate/hydroxycinnamoyl transferase from *Avena sativa* (BAC78633), CcsHQT1: HQT from *C. cardunculus* var. *scolymus* (CAM84302), CcsHQT2: HQT from *C. cardunculus* var. *scolymus* (CAR92145), CcaHQT: HQT from *C. cardunculus* var. *altilis* (ABK79690), CiHQT1: this work (KT222893), CiHQT2: this work (KT222894), CiHQT3: this work (KT222895), CcHQT: HQT from *C. canephora* (ABO77957), NtHQT: HQT from *N. tabacum* (CAE46932), SlHQT: HQT from *Solanum lycopersicum* (CAE46933), SsHCT: HCT from *Solenostemon scutellarioides* (CAK55166), LaAT1: alcohol acyltransferase 1 from *Lavandula angustifolia* (ABI48360), DcHCBT: anthranilate *N*-hydroxycinnamoyl/benzoyl transferase from *Dianthus caryophyllus* (CAB06430), TpHCT2: HCT from *Trifolium pratense* (ACI16631), OsHCT3: Hydroxycinnamoyl shikimate/glycerol transferase from *O. sativa* (NP_001056998), OsHCT4: Hydroxycinnamoyl shikimate/glycerol transferase from *O. sativa* (NP_001057003), AtSHT: spermidine *N*-hydroxycinnamoyl transferase from *A. thaliana* (AEC06845), MdSHT: spermidine *N*-hydroxycinnamoyl transferase from *Malus domestica* (ALF00095).

Phylogenetic analysis of predicted amino acid sequences confirm our first analysis, i.e., HCT1 and HCT2 group together with biochemically characterized HCTs whereas HQT1, HQT2, and HQT3 group with characterized HQTs (**Figure 5A**). All five proteins contain the two conserved motifs, HXXXDG and DFGWG that are observed among BAHD acyltransferases (**Figure 5B**). The first one, implicated in the active site, is strongly conserved whereas the second one could be slightly altered. HCT1 and HCT2 contain the same motif HHAADG in the middle part of the protein whereas HQT1 and HQT2 share the motif HTLSDG. For HQT3, a unique motif HTLADG was found. In the C-terminal part of the proteins, all proteins harbor the same DFGWG motif except HQT2 which amino sequence is DFGYG. Sequencing and analyses of genomic sequences show that *HCT1*, *HQT1, HQT2*, and *HQT3* exhibit a single intron whereas *HCT2* has none. The introns are 242, 1203, 1293, and 873 bp in length for *HCT1*, *HQT1, HQT2*, and *HQT3*, respectively. They harbor the features of the conserved intron “Q” found in many members of the BAHD family (St-Pierre and De Luca, 2000). The intron position in the coding region corresponds to an insertion between a Gln residue and a Val residue of the predicted amino acid sequences (**Figure 5B**).

### Expression of HCTs and HQTs in *E. coli* and Evaluation of Their Activity *In Vitro*

**FIGURE 6:**
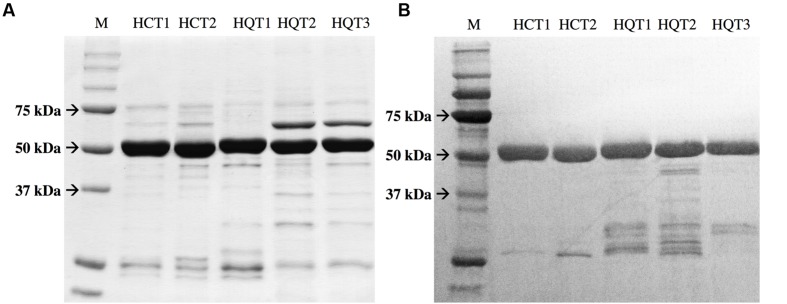
**Expression and purification of recombinant HCT and HQT proteins. (A)** SDS-PAGE separation of recombinant HCT and HQT. The gel was stained with Coomassie blue. **(B)** Immunoblot analysis confirming the expression of the recombinant proteins. The proteins were detected with anti-His antibody. M: Markers.

**FIGURE 7:**
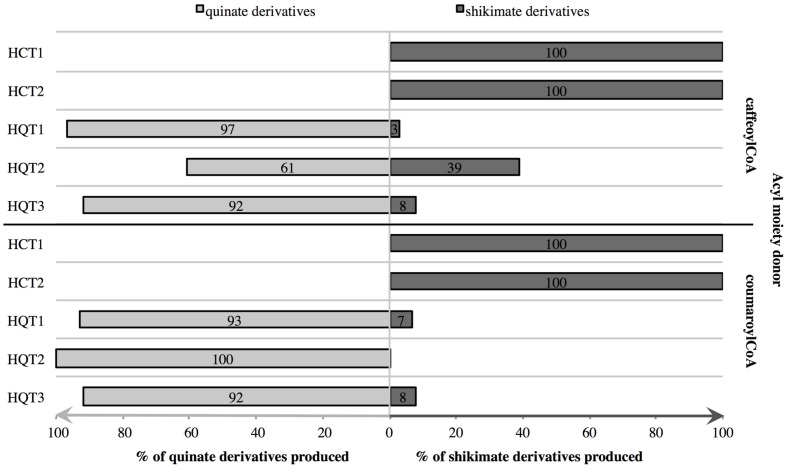
**Competition assays to determine the preferred acyl acceptor of HCTs and HQTs**. Recombinant proteins were incubated with caffeoyl-CoA or *p*-coumaroyl-CoA and both quinic and shikimic acids at the same concentrations. After 1 h, products of the reactions were quantified in μmol and the relative concentrations of each product calculated (% of total products). Values are means of three independent replicates.

To study the catalytic activities of the chicory HCT and HQT proteins and to confirm the phylogenetic analysis, their entire coding regions were cloned into pDEST-17 vectors for expression in *E. coli*. Proteins were recovered in the soluble fractions and purified using His-Trap Ni columns. Presence, identity and purity of the recombinant proteins were then validated by SDS-PAGE and by immunoblot analysis using anti-His antibody (**Figure 6**). All proteins give a single band at the expected molecular mass, i.e., about 50 kDa (His_6_-HCTs or His_6_-HQTs). Activities of recombinant proteins were measured *in vitro* using either *p*-coumaroyl-CoA or caffeoyl-CoA as acyl donor and quinate or shikimate as the acceptor of the transferase reaction. The products of the reaction were analyzed by HPLC. In the presence of the enzymes, coumaroylquinate, coumaroylshikimate, caffeoylquinate, and caffeoylshikimate were detected in the reaction mixtures containing different combinations of substrates (not shown). No product could be detected in the absence of proteins or in the absence of the free acid. To verify their substrate preference, reaction mixtures containing 5 mM quinic acid plus 5 mM shikimic acid were run in the presence of 0.4 mM caffeoyl-CoA or 0.4 mM *p*- coumaroyl-CoA. When both acids were provided at the same concentration, HCTs only produce caffeoylshikimate or coumaroylshikimate whereas HQTs produce predominantly caffeoylquinate or coumaroylquinate (**Figure 7**). These results clearly demonstrate that HCTs from chicory strongly prefer shikimic over quinic acid whereas HQTs prefer quinic acid. These biochemical data support the phylogenetic tree and are in accordance with previously published reports dealing with HCTs or HQTs from other species. In additional experiments, we have shown that HCTs and HQTs can also use feruloyl-CoA and cinnamoyl-CoA as acyl donors but at much smaller rates (not shown). The activity of HCTs and HQTs toward other potential substrates (spermidine, tartaric acid) was also tested but no activity was observed in our experimental conditions.

These reactions were shown to be fully reversible in other systems (Hoffmann et al., 2003; Comino et al., 2007, 2009). In our experiments, when recombinant HCTs or HQTs were incubated in the presence of 3-CQA and CoA, caffeoyl-CoA was detected except for HCT2 (not shown).

The optimal pHs of HCT1 and HQT1 were evaluated in the presence of caffeoyl-CoA and shikimate or quinate, respectively (Supplementary Figure S1). Optimum pHs are 7.5 for HCT1 and 5.0 for HQT1. Activity of HCT1 readily decreases at acidic or alkaline pHs. At pH 6.5 and 8.0, reaction rates are 61%. For HQT1, the activity slowly declines at alkaline pHs. At pH 7.0, activity is still 60% whereas at acidic pH, reaction rates rapidly decline (21% at pH 4.0).

### Kinetic Parameters of Recombinant Proteins

Kinetic parameters of the enzyme were determined using caffeoyl-CoA or *p*-coumaroyl-CoA as the acyl-CoA donors and either quinate for HQTs or shikimate for HCTs as acyl acceptors (**Table 1**). The reactions showed typical Michaelis-Menten kinetics with increasing concentrations of acyl acceptor. The K_m_ values and V_max_ values were calculated from triplicates by the Lineweaver–Burk method. As shown in **Table 1A**, HCTs have better affinity for shikimate in the presence of saturating concentration of *p*-coumaroyl-CoA than in the presence of saturating concentration of caffeoyl-CoA (for HCT1, K_m_ = 320 ± 40 and 8000 ± 600 μM, respectively). On the contrary, HQT1 has better affinity for quinate in the presence of caffeoyl-CoA (**Table 1B**, K_m_ = 160 ± 34 μM with caffeoyl-CoA and 3800 ± 413 μM with *p*-coumaroyl-CoA). HQT2 and HQT3 behave similarly and have about the same affinity for quinate in the presence of either caffeoyl-CoA or *p*-coumaroyl-CoA.

**Table 1:** Kinetic parameters of HCTs and HQTs from chicory.

(A) HCT1 and HCT2.							
**Varying substrate**	**Saturating substrate**	**HCT1**			**HCT2**		
		**K_m_ ± SD**	**V_max_ ± SD**	**V_max_/K_m_**	**K_m_ ± SD**	**V_max_ ± SD**	**V_max_/K_m_**
		**μM**	**nkat mg^-1^**	**nkat mg^-1^μM^-1^**	**μM**	**nkat mg^-1^**	**nkat mg^-1^μM^-1^**

Shikimate	*p*-coumaroyl-CoA	320 ± 40	237 ± 9	0.74	802 ± 63	303 ± 9	0.38
Shikimate	Caffeoyl-CoA	8000 ± 600	618 ± 34	0.08	5080 ± 360	695 ± 31	0.14

**(B) HQT1, HQT2, and HQT3**.										

**Varying substrate**	**Saturating substrate**	**HQT1**			**HQT2**			**HQT3**		
		**K_m_ ± SD**	**V_max_ ± SD**	**V_max_/K_m_**	**K_m_ ± SD**	**V_max_ ± SD**	**V_max_/K_m_**	**K_m_ ± SD**	**V_max_ ± SD**	**V_max_/K_m_**
		**μM**	**nkat mg^-1^**	**nkat mg^-1^ μM^-1^**	**μM**	**nkat mg^-1^**	**nkat mg^-1^ μM^-1^**	**μM**	**nkat mg^-1^**	**nkat mg^-1^ μM^-1^**

Quinate	*p*-coumaroyl-CoA	3800 ± 413	903 ± 49	0.24	637 ± 65	149 ± 6	0.23	416 ± 240	74 ± 11	0.18
Quinate	Caffeoyl-CoA	162 ± 34	113 ± 6	0.70	613 ± 53	134 ± 4	0.22	318± 71	165± 10	0.52

*The Michaelis constants (K_*m*_) were determined using various acid concentrations and saturating concentrations of acyl-CoA (800 μM). Data are summarized as the arithmetic mean ± SD of three independent experiments*.

### Chicory HCTs and HQTs Are Differentially Expressed

The expression pattern of chicory *HCT*s and *HQT*s were examined using quantitative real-time PCR (qRT-PCR) in different parts of chicory seedlings shown to accumulate contrasted levels of phenolic compounds and in culture cells treated or not with MeJA. Primers were design to detect specifically *HCT1*, *HCT2*, *HQT1*, *HQT2*, or *HQT3*. The expression data of the target genes were normalized to *CLATH* and *SAND* mRNA levels for seedling experiments and to *TIP41* and *PP2AA2* mRNA levels for cell culture experiments in agreement with previous work done on the same plant material (Delporte et al., 2015).

**FIGURE 8:**
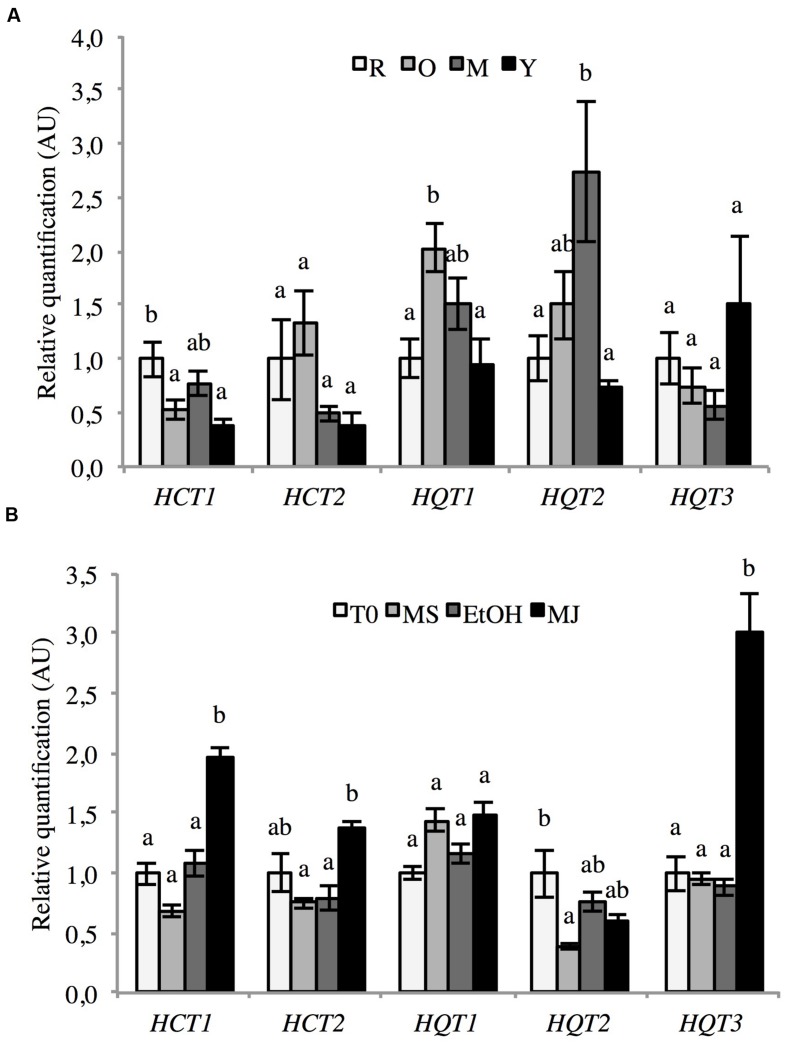
**Expression of HCT1, HCT2, HQT1, HQT2, and HQT3 in various chicory tissues**. mRNA abundance in different parts of 6-weeks-old seedlings **(A)** and culture cells elicited or not with MeJA **(B)** was determined by qRT-PCR. **(A)** Expression levels were normalized to the expression of CLATH and SAND and presented relative to that of Roots. Y: young leaves, M: intermediate leaves, O: Old leaves, R: roots. **(B)** Expression levels were normalized to the expression of TIP41 and PP2AA2 and presented relative to that of T0. T0: cells sampled before elicitation, MS: cultures kept on MS medium for 24 h, EtOH: cell cultures treated with ethanol for 24 h, MJ: cell cultures treated with 50 μM MeJA for 24 h. Error bars indicate SEM. Different letters above histograms indicate statistical difference highlighted using ANOVA (*P*-value < 0.05).

The five genes were expressed in all of the investigated tissues (roots, old leaves, intermediate leaves, and young leaves; **Figure 8A**). Each gene shows a tissue-specific pattern of accumulation. Nevertheless no significant difference could be detected. HCT1 is the most expressed in roots whereas HCT2 and HQT1 expressions are the highest in the old leaves. HQT2 and HQT3 expressions are the highest in the intermediate leaves and in the youngest leaves, respectively.

In cell culture, MeJA treatment up-regulates the mRNA abundance of *HCT1*, *HCT2*, and *HQT3* (**Figure 8B**). For these three genes, mRNA abundance in MeJA treated cells was about 2-, 1.5-, and 3-fold higher respectively than in control cells. The expression of the two other acyltransferases (*HQT1* and *HQT2*) was rather stable in all investigated conditions.

### Functional Analysis of HCT1 and HQT1

**FIGURE 9:**
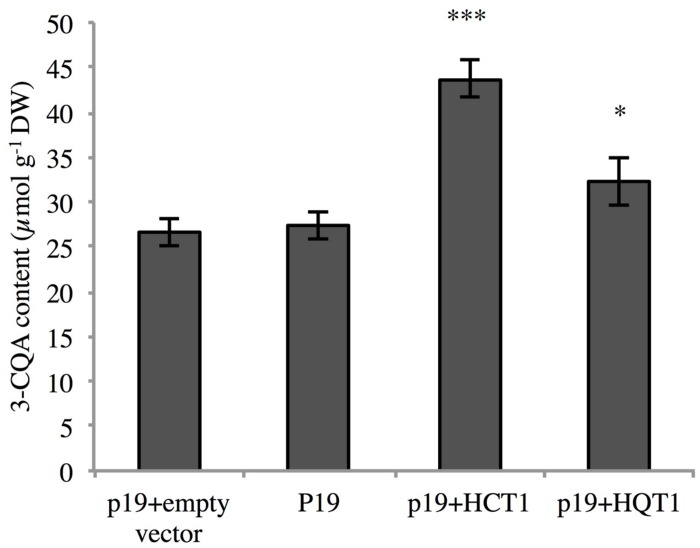
**Determination of CQA content in *Nicotiana benthamiana* transiently transformed leaves**. Transformants either carried the pEAQ-HT vector (p19) alone or associated with the empty pB2GW7 vector (p19+empty vector) or with the pB2GW7-HCT1 vector (p19+HCT1- or with the pB2GW7-HQT1 vector (p19+HQT1). The data represent means ± SEM of eight transiently transformed leaves from four independent plants. Statistical differences were highlighted compared to the control p19+empty vector using one tail Student’s test (^∗^*P* < 0.05; ^∗∗∗^*P* < 0.001).

In order to assess the function of HCTs and HQTs *in planta*, HCT1 and HQT1 were transiently expressed in *N. benthamiana*, a species known to accumulate large amounts of CQA (Niggeweg et al., 2004). *N. benthamiana* plants were co-infiltrated with *A. tumefaciens* containing constructs of HCT1 (or HQT1) or the p19 gene. As control, leaves were infiltrated with the p19 vector only or the empty pB2GW7 vector (see Materials and Methods for the details). After 4 days, leaves were collected and the amounts of CQA measured. 3-CQA was the most abundant CQA isomer in leaves of tobacco. In leaves transiently expressing HQT1 or HCT1, levels of 3-CQA were higher than in leaves infiltrated with the empty vector or the p19-harboring plasmid (**Figure 9**). The increase of 3-CQA levels was more pronounced in HCT1 inoculated plants. Leaves infiltrated with the empty vector or the p19-harboring vector accumulate similar levels of 3-CQA.

## Discussion

Hydroxycinnamoyl esters play essential roles in plant physiology especially in plant-environment interactions (Sullivan, 2014). Furthermore, these compounds have significance in the area of human health. They are present in fruits and vegetables and they represent important antioxidant molecules with multiple applications for cosmetic, pharmaceutical and food industries. Industrial chicory accumulates four main caffeic esters, i.e., 3-CQA, 3,5-diCQA, CTA, and diCTA (this work; Kandeler and Ullrich, 2009; Bahri et al., 2012). An additional CQA isomer, i.e., 5-CQA known as neochlorogenic acid was also detected in root tissues (this work). Its content was quite low in seedling roots (0.0035% DW, i.e., 37 times lower than 3-CQA content in the same tissue) but was significantly higher in root cell cultures (from 0.088 to 0.156% DW in our set of experiments). These molecules exhibit an original tissue distribution pattern. Tartaric acid derivatives are predominantly accumulated in aerial parts and diCQA is mainly located in roots. The contents of 3-CQA is equally distributed between organs whereas 5-CQA, although at low level, is exclusively located in roots. We presume that these specific localizations must rely on specific tissue functions that largely remain to be elucidated. In addition, detailed analysis of aerial parts revealed that contents of all phenolic acids were related to leaf age. Young leaves accumulate the highest levels of CTA, diCTA, 3-CQA, and diCQA whereas oldest leaves accumulate the lowest levels of these compounds. Total level of molecules reaches 56 μmol g^-1^ DW in the youngest expanding leaves whereas levels were 26 and 16 μmol g^-1^ DW in intermediate leaves and in oldest leaves, respectively. This could suggest a role of these compounds in biotic or abiotic stress protection, these tissues being the most valuable parts of the plants. This is in favor of the so-called optimal defense theory. This theory suggests that plants accumulate more constitutive defense metabolites in tissues that are crucial in the fitness of the plant (McKey, 1974; Rhoades, 1979). Notably CQA was shown to have a role in plant protection against thrips in Chrysanthemum (Leiss et al., 2009). This theory was already suggested to explain the original tissue pattern accumulation of phenolamides (phenylpropanoid-polyamine conjugates) in *Nicotiana attenuata* (Kaur et al., 2010). These phenylpropanoid derivatives were preferentially allocated to the young leaves presumably to prevent pathogen or herbivore attacks. Whether, this original pattern of accumulation in chicory seedlings relies on more active synthesis of these molecules in young expanding leaves, more active catabolism of these molecules in oldest leaves or more efficient transport of phenolics to the growing tissues still remain to be determined.

To take advantage of this original chemical composition (traditional breeding, synthetic biology, genetic engineering), a full understanding of the relevant biosynthetic pathways is required. Evidences in other species prompted us to focus our analysis on the clade Vb of the BAHD superfamily of plant-specific acyl-CoA dependent acyltransferases (St-Pierre and De Luca, 2000). This clade was shown to contain proteins involved in the synthesis of CQA and related compounds as well as more diverse products like phaselic acid, triferuloyl spermidine and feruloyl glycerol (Grienenberger et al., 2009; Sullivan and Zarnowski, 2011; Kim et al., 2012; Elejalde-Palmett et al., 2015). The full-length open reading frames of five candidate genes were cloned. On the basis of their high similarity with the already functionally characterized hydroxycinnamoyl-transferase sequences (HCT or HQT) and of the phylogenetic analysis, they were named *HCT1*, *HCT2*, *HQT1*, *HQT2*, and *HQT3*. They share typical features of the members of the BAHD family (D’Auria, 2006). Phylogenetic clustering can give a clue of the function. Nevertheless function need to be established on a biochemical demonstration of activity. Clade Vb of the BAHD contain members with very versatile catalytic specificities (Grienenberger et al., 2009; Sullivan and Zarnowski, 2011; Kim et al., 2012; Elejalde-Palmett et al., 2015). All five proteins were shown to be able to use either *p*-coumaroyl-CoA or caffeoyl-CoA as an acyl donor and quinic acid or shikimic acid as an acyl acceptor. Substrate specificities and associated kinetic parameters are consistent with those of HCTs or HQTs isolated and cloned in other species (Hoffmann et al., 2003; Niggeweg et al., 2004; Sonnante et al., 2010). Abilities to catalyze the reverse reactions were also examined and confirmed for four of the five enzymes as found in other species.

Phenylpropanoid contents usually rise when the plant is submitted to MeJA elicitation (Gális et al., 2006). We found out that treatment of chicory cell cultures with MeJA leads to an increase of 3-CQA and 3,5-diCQA amounts. Furthermore, in contrast to root or leaf extract where only one major isomer of CQA (i.e., 3-CQA) could be detected, in suspension culture, 5-CQA was detected in equivalent quantity to 3-CQA. We observed that treatment of cell cultures with MeJA induces the expression of the genes encoding HQT3 and the two HCTs with a stronger induction for *HCT2* and even more for *HQT3*. This suggests the major involvement of HQT3 in the higher production and accumulation of 3-CQA in response to MeJA treatment. No relationship between 3-CQA or 3,5-diCQA accumulation in young leaves (compare to the oldest leaves) and HCT or HQT expression was shown.

The involvement of HQTs and HCTs in 3-CQA production was further confirmed by *in vivo* functional analysis. HCT1 and HQT1 were transiently overexpressed in tobacco. The results clearly demonstrate that HCT1 and HQT1 are involved in the synthesis of CQA. This is in accordance with previous studies dealing with HQT from artichoke, tobacco, or tomato overexpressed transiently or stably in tobacco (Niggeweg et al., 2004; Sonnante et al., 2010). Surprisingly, HCT1 transient expression promoted the accumulation of 3-CQA at higher level than that of HQT1. Considering substrate affinity of these two enzymes *in vitro*, we are prone to suggest that *in planta* pathway for 3-CQA production toward its accumulation occurs in two steps. First caffeoyl-CoA is synthesized through the successive action of HCT, C3′H and either HCT or HQT (reverse reaction). Subsequently, HQT uses the CoA ester to produce 3-CQA. This assumption is also supported by the transcriptional analysis. Indeed 3-CQA accumulation promoted by MeJA induction is concomitant to an increase of the expression of HCTs and HQT3. If HQT was the only required enzymatic step, one could expect the induction of the sole HQT expression. This route for the synthesis of CQA was already favored in artichoke (Sonnante et al., 2010). Docking and modeling experiments showed that HQTs preferred quinate as a substrate whereas HCTs preferred shikimate. Better efficiency of artichoke C3′H in the conversion of coumaroylshikimate into caffeoylshikimate also favored this hypothesis (Moglia et al., 2009). Preference of HCTs for shikimate and coumaroyl-CoA was also demonstrated by structural analysis in *Sorghum bicolor* (Walker et al., 2013). SbHCT activity was proven to be quite restricted to the production of coumaroylshikimate. All together these data infer that esterification by HCT should be the limiting catalytic step in CQA synthesis. Flux analysis should confirm this assumption. Alternatively, CQA synthesis promoted by HQT could be limited by substrate availability due to HQT specific subcellular localization. Optimal pH of this enzyme was shown to be around 5. This could fit with a vacuolar compartmentalization of this enzyme. Such localization of HQT was already shown in *S. lycopersicum* (Moglia et al., 2014). In this context, HQT activity rate could be limited by CoA-ester or quinic acid import into vacuole.

The presence of several homologous genes might relate various independent separations of them during plant evolution. Therefore, in chicory, HQT enzymes and HCT enzymes seem to be encoded by a gene family of at least three members and two members, respectively. This seems to be a general trend in the *Asteraceae* family since three HQTs and one HCT were found in artichoke and several members of each family are present in sunflower and in lettuce (Sonnante et al., 2010). The presence of multiple isoforms of each family might ensure an optimization of fluxes toward the accumulation of different metabolites. Especially, in chicory, in addition to CQA accumulation, CQA might serve as a caffeoyl donor for the production of diCQA, CTA, and diCTA. Further experiments are needed to decipher the physiological roles of these enzymes. Tissue and subcellular localization of the products of these genes could provide clues of their function.

This work is the first contribution in the understanding of the genetic basis of the hydroxycinnamate biosynthesis in *C. intybus*. Future research will be devoted to extend the investigation on CQA accumulation but also to the characterization of the CTA and diCTA biochemical pathway as well as that of the diCQA, which remains incomplete at least in the *Asteraceae*.

## Author Contributions

GL, MD, CK, MM, and DG carried out the molecular genetic studies. AH, CV, and PH set up the cell culture system and performed preliminary experiments. GL, MD, CK, and DG carried out the biochemical experiments. DG and J-LH planned and supervised the work. DG wrote the article with contributions of all the authors. All authors read and approved the final manuscript.

## Conflict of Interest Statement

The authors declare that the research was conducted in the absence of any commercial or financial relationships that could be construed as a potential conflict of interest.
