# Supplementary material for: Identification and Characterization of Five BAHD Acyltransferases Involved in Hydroxycinnamoyl Ester Metabolism in Chicory
Source: Front Plant Sci. 2016 Jun 6;7:741. doi: 10.3389/fpls.2016.00741 (PMC4893494; doi:10.3389/fpls.2016.00741)
Supplement: Supplementary file 1 [file Table_1.PDF]

**Supplemental Table S1: Sequence of primers used in this study.** This includes oligonucleotides used for cloning and expression analysis. CLATH: Clathrin adaptator complex subunit, TIP41: TIP41 like protein, PP2AA2: Protein Phosphatase 2A subunit A2, SAND: SAND family protein.

| Primer sequence (5' to 3') | | Gene | Application |
|---|---|---|---|
| CACACATGAAGATCGAGGTGAGAGA | Fwd | HCT1 | Subcloning into pGEM-T easy, genomic DNA cloning |
| CACACTTATATATCATAAAGAAACTTGCTAA | Rev | | |
| CACACATGACTAACGGAGCTGGTTC | Fwd | HQT1 | |
| CACACCAAGACTCTAAAAGTCGTACAAGT | Rev | | |
| CACACATGAAGATCGCGATTAGAGAAT | Fwd | HCT2 | |
| CCAACATTTCAGATATCATACAAGA | Rev | | |
| CGATGAGAAGTGATCAAAAGATG | Fwd | HQT2 | |
| CCTTACATTTCATACAAAAACTTCTCG | Rev | | |
| CACACATGGGGAGCGATCACAAA | Fwd | HQT3 | |
| CACACTTAAAACTCATACAAGAACTTCTCAAA | Rev | | |
| GGGGACAAGTTTGTACAAAAAAGCAGGCTCCATGACTAACGGAGCTGGTTCG | Fwd | HQT1 | Cloning into pDEST17 |
| GGGGACCACTTTGTACAAGAAAGCTGGGTCCTAAAAGTCGTACAAGTACTTTTGGA | Rev | | |
| GGGGACAAGTTTGTACAAAAAAGCAGGCTCCATGAAGATCGAGGTGAGAGAATC | Fwd | HCT1 | |
| GGGGACCACTTTGTACAAGAAAGCTGGGTCTTATATATCATAAAGAAACTTGCTAAAAACC | Rev | | |
| GGGGACAAGTTTGTACAAAAAAGCAGGCTCCATGAAGATCGCGATTAGAGAATC | Fwd | HCT2 | |
| GGGGACCACTTTGTACAAGAAAGCTGGGTCTCAGATATCATACAAGAACTTGCTAAA | Rev | | |
| GGGGACAAGTTTGTACAAAAAAGCAGGCTCCATGAGAAGTGATCAAAAGATGATGAT | Fwd | HQT2 | |
| GGGGACCACTTTGTACAAGAAAGCTGGGTCTTACATTTCATACAAAAACTTCTCGAA | Rev | | |
| GGGGACAAGTTTGTACAAAAAAGCAGGCTCCATGGGGAGCGATCACAAA | Fwd | HQT3 | |
| GGGGACCACTTTGTACAAGAAAGCTGGGTCTTAAAACTCATACAAGAACTTCTCAAA | Rev | | |
| TTGCAGGTGAGCTAATGTCG | Fwd | HCT1 | qRT-PCR |
| CGAACCAGAGCCTTCAAATC | Rev | | |
| TGGGCTAGGCTTCCTATTCA | Fwd | HCT2 | |
| AAATGGCAATCCACAAGCTC | Rev | | |
| CCCCACCATCACTAATCACC | Fwd | HQT1 | |
| CGGCTATGATCACGAATGTG | Rev | | |
| TTGGAGTCGCAAAAGGATCT | Fwd | HQT2 | |
| GCCATATCCGAAATCAGCAT | Rev | | |
| CGACTCAACCGCTTTCAAAT | Fwd | HQT3 | |
| ACTCGGACCACGAATCAAAG | Rev | | |
| TGCTTCCGCCATCTACTTTT | Fwd | CLATH | |
| TCCCAAGTTCCTTTGTTTGC | Rev | | |
| GTTGGGTGCGCATCTCTAAT | Fwd | TIP41 | |
| AGCTCCGGCAGCTTTTACTT | Rev | | |
| CATGGGCTCAGAAATCACCT | Fwd | PP2AA2 | |
| ATTGGTCAACGATGGGGATA | Rev | | |
| TGCTTACACCACAAGGCAAG | Fwd | SAND | |
| GAAGCAGCATGTCATCAGGA | Rev | | |
| GGGGACAAGTTTGTACAAAAAAGCAGGCTCCACCATGAAGATCGAGGTGAGAGAATC | Fwd | HCT1 | Cloning into pB2GW7 |
| GGGGACCACTTTGTACAAGAAAGCTGGGTCTTATATATCATAAAGAAACTTGCTAAAAACC | Rev | | |
| GGGGACAAGTTTGTACAAAAAAGCAGGCTCCACCATGACTAACGGAGCTGGTTCG | Fwd | HQT1 | |
| GGGGACCACTTTGTACAAGAAAGCTGGGTCCTAAAAGTCGTACAAGTACTTTTGGA | Rev | | |
