# Supplementary material for: Identification and Characterization of Five BAHD Acyltransferases Involved in Hydroxycinnamoyl Ester Metabolism in Chicory
Source: Front Plant Sci. 2016 Jun 6;7:741. doi: 10.3389/fpls.2016.00741 (PMC4893494; doi:10.3389/fpls.2016.00741)
Supplement: Supplementary file 2 [file Table_2.PDF]

Supplemental Table S2: Caffeic acid ester contents in leaves and roots of 6-week-old plants of chicory. Values are means of 4 independent biological replicates ± SD. nd = Not detected.

	Roots		Leaves	
	μmol g^{-1} DW	% Total	μmol g^{-1} DW	% Total
**Chlorogenic acid (3-CQA)**	3.7 ± 1.6	34.6	6.1 ± 1.2	20.5
**Neochlorogenic acid (5-CQA)**	0.1 ± 0.0	0.9	nd	0
**Isochlorogenic acid (3,5-diCQA)**	6.7 ± 3.0	62.6	0.3 ± 0.1	1.0
**Caftaric acid (CTA)**	nd	0	4.5 ± 0.8	15.1
**Chicoric acid (diCTA)**	0.2 ± 0.1	1.9	18.9 ± 2.2	63.4
**Total**	**10.7± 4.7**	**100**	**29.8 ± 4.3**	**100**
