# Supplementary material for: Identification and Characterization of Five BAHD Acyltransferases Involved in Hydroxycinnamoyl Ester Metabolism in Chicory
Source: Front Plant Sci. 2016 Jun 6;7:741. doi: 10.3389/fpls.2016.00741 (PMC4893494; doi:10.3389/fpls.2016.00741)
Supplement: Supplementary file 3 [file Image_1.PDF]

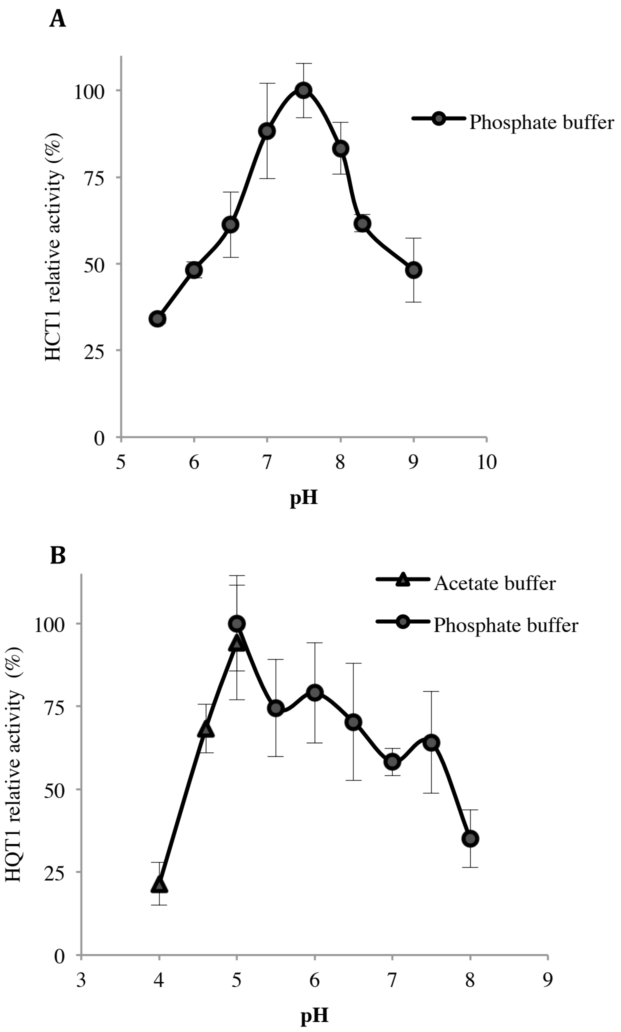

Supplemental Figure S1. Effect of the pH on the activity of the recombinant proteins. A: HCT1, B: HQT1. Activities were measured in phosphate buffer (circles) or acetate buffer (squares) and are expressed as percentage of the activity measured at optimal pH. HCT1 activities were measured in the presence of caffeoyl-CoA and shikimate. HQT1 activities were measured in the presence of caffeoyl-CoA and quinate. Values are mean of three replicates ± SD.
